# A FRET-Based Biosensor for the Src N-Terminal Regulatory Element

**DOI:** 10.3390/bios12020096

**Published:** 2022-02-04

**Authors:** Guillermo Iruela, Alejandro Fernández, Amin Sagar, Francisco Javier Carvajal, Pau Bernadó, Miquel Pons

**Affiliations:** 1 BioNMR Laboratory, Department of Inorganic and Organic Chemistry, Universitat de Barcelona, Baldiri Reixac, 10-12, 08028 Barcelona, Spain; giruela20@gmail.com (G.I.); alejandrofernandez@ub.edu (A.F.); frjcarvajal@gmail.com (F.J.C.); 2 IDP Discovery Pharma, S.L. Barcelona Science Park, Baldiri Reixac, 10-12, 08028 Barcelona, Spain; 3 Centre de Biologie Structurale (CBS), Université de Montpellier, CNRS, Inserm 29, rue de Navacelles, 34090 Montpellier, France; amin.sagar@cbs.cnrs.fr (A.S.); pau.bernado@cbs.cnrs.fr (P.B.)

**Keywords:** intrinsically disordered proteins (IDP), fuzzy complexes, high-throughput screening, c-Src, fluorescence, NMR

## Abstract

In signaling proteins, intrinsically disordered regions often represent regulatory elements, which are sensitive to environmental effects, ligand binding, and post-translational modifications. The conformational space sampled by disordered regions can be affected by environmental stimuli and these changes trigger, vis a vis effector domain, downstream processes. The disordered nature of these regulatory elements enables signal integration and graded responses but prevents the application of classical approaches for drug screening based on the existence of a fixed three-dimensional structure. We have designed a genetically encodable biosensor for the N-terminal regulatory element of the c-Src kinase, the first discovered protooncogene and lead representative of the Src family of kinases. The biosensor is formed by two fluorescent proteins forming a FRET pair fused at the two extremes of a construct including the SH4, unique and SH3 domains of Src. An internal control is provided by an engineered proteolytic site allowing the generation of an identical mixture of the disconnected fluorophores. We show FRET variations induced by ligand binding. The biosensor has been used for a high-throughput screening of a library of 1669 compounds with seven hits confirmed by NMR.

## 1. Introduction

Intrinsically disordered regions (IDR) are a common, yet poorly understood, feature of eukaryotic proteins [1,2]. Although proteins with large disordered regions represent around two thirds of the proteins of humans, and other eukaryotes, they are far less abundant in bacteria and archaea [3]. This distribution suggests an important role in organisms requiring much more complex regulation [4]. Indeed, the analysis of IDP functions shows an enrichment in proteins whose disfunction is linked to cancer, neurodegenerative or cardiovascular diseases [5].

Intrinsically disordered regions do not adopt a single static conformation but exist in their native form as an ensemble of rapidly exchanging forms. Their lack of a static three-dimensional structure does not prevent function, challenging the classical structure-function paradigm [6] and the classical structure-based drug design [7]. Initially considered undruggable targets, due to the absence of a well-defined binding site, this notion has also been superseded by the recent discovery of drugs acting on intrinsically disordered proteins [8,9,10].

Src is the leading member of the Src family of tyrosine kinases (SFK) formed by 9 members in humans: Src, Yes, Fyn, Fgr, Blk, Hck, Lck, Lyn, and Frk. All of them are non-receptor tyrosine kinases implicated in various aspects of cell signaling. Src itself regulates cell growth, survival, migration and invasion [11,12]. High levels of Src activity are associated with poor prognosis in multiple cancer types [13,14,15,16]. All SFK members share a common domain architecture formed, starting from the N-terminus, by the SH4, unique, SH3, SH2 and SH1 (or kinase) domains. The SH3, SH2 and SH1 domain are globular, folded domains and their sequences and structures are well conserved among SFKs. In contrast, the SH4 and unique domains are intrinsically disordered and show very low sequence conservation among the various SFK proteins, although the sequences of the corresponding domains in the same proteins of various species are conserved by evolution, indicating that they are functionally important. Our group has demonstrated that localized mutations in the unique domain result in 50% decrease in the invasive capacity of Src-dependent colorectal cancer cell lines [17] validating this intrinsically disordered region as a potential drug target.

The Src SH4 and unique domains, while remaining disordered, are dynamically interacting with the SH3 domain. The rapidly interchanging weak interactions between the disordered regions and the scaffolding globular domain define an intramolecular fuzzy complex [17,18]. This fuzzy complex is thought to be the functional unit transferring the signals “sensed” by the disordered regions to the globular domains and, in particular, the kinase domain that will transfer the information downstream through a phosphorylation cascade. The functional unit formed by the SH4, unique, and SH3 domains is referred to as the Src N-terminal regulatory element (SNRE). Although the details of the connection between the SNRE and the rest of the protein remain obscure, we decided to design a biosensor directly monitoring changes in the SNRE caused by changes in the environment, such as ligand binding. We reasoned that the end-to-end distance between the extremes of the SNRE could be a sensitive reporter of changes in the fuzzy complex and we fused a green fluorescent protein (mClover3) to the N-terminus and a red protein (mRuby3) to the C-terminus of the SH3 domain [19,20]. The biosensor was dubbed CLOBY indicating the identity of the CLOver and ruBY fluorescent proteins. These two proteins form an efficient Förster Resonance Energy Transfer (FRET) pair in which the energy absorbed by the green protein is non-radiatively transferred to and emitted by the red protein with an efficiency that decreases with the inverse sixth power of the distance. The biosensor containing the fluorescent proteins, the SNRE (144 residues), suitable spacers (16 residues) and a purification tag, are expressed by a single synthetic gene enabling the expression of the biosensor in cells. Indirectly monitoring perturbations in an intrinsically disordered regions through changes in the end-to-end distance sensed by FRET represents a novel approach for high-throughput screening of drugs targeting disordered domains.

Here we report that the fluorescence of CLOBY is affected by the binding of the VSL12 peptide [21], a known ligand of the SNRE [18], and an in vitro high-throughput screening of a chemical library of 1669 compounds that identified seven hits confirmed by NMR.

## 2. Materials and Methods

*SNRE biosensor.* A synthetic gene encoding CLOBY with codons optimized for its expression in *Escherichia coli* and inserted into an expression vector PET19b-X was obtained from ATG:biosynthetics. The construct contains a six-histidine tag at its N-terminus to facilitate its purification by a nickel affinity column. The protein was expressed in *E. coli* BL21(DE3) cells.

Transformed cells were precultured in 10 mL of Luria Broth supplemented with ampicillin overnight at 37 °C and then transferred to 1 L cultures in a 3 L Erlenmeyer flask until an optical density at 600 nm of 0.5–0.8 was obtained. Expression was induced by 1 mM isoproylthiogalactoside (IPTG) for 14–18 h at 25 °C followed by 36 h at 20 °C. Continuous agitation at 120 rpm ensured the proper aeration needed for cell growth and, importantly, for subsequent maturation of the fluorescent proteins.

Cell pellets were collected by centrifugation at 5000 rpm for 15 min, resuspended in cell lysis buffer (20 mM Tris, 300 mM NaCl, 10 mM imidazole, 0.01% NaN_3_, pH 8) supplemented with protease inhibitor cocktail (0.23 mM 4-(2-aminoethyl)benzenesulfonyl fluoride (AEBSF), 20 μM Bestatin, 1 mM EDTA, 3 μM E64, 3 uM Pepstatin A) and 1 mM phenylmethysulfonyl fluoride (PMSF), frozen to −80 °C, thawed to 4 °C and lysed by sonication in an ice bath followed by a treatment with DNaseI for 15 min at 4 °C. The supernatant after centrifugation at 25,000 rpm for 20 min, was purified by nickel affinity (HiTrap, GE Healthcare), washed with lysis buffer (10 mM imidazole) and eluted with 400 mM imidazole. Final purification was achieved by size-exclusion chromatography using a Superdex 75 26/60 column or a Superdex 200 column (GE Healthcare) eluted with 50 mM sodium phosphate, 150 mM NaCl, 200 μM EDTA, 0.01% NaN_3_, pH 7.5). The correct molecular weight of CLOBY, after the maturation of both chromophores is 72,639 Daltons, confirmed by mass spectrometry.

A side effect of incomplete maturation of the red protein is a truncated protein that is removed by size-exclusion chromatography. Final purity is checked by SDS-PAGE. A variable proportion of non-matured red protein is present in the final samples. An estimation of the degree of maturation of the red chromophore can be obtained by comparing the concentrations determined using the absorbance at 280 nm, 506 nm (mClover3), and 558 nm (mRuby3). The lowest concentration is observed at 558 nm and corresponds to the fully mature protein that typically represents (50–70%) of the total protein. The degree of maturation is not constant among preparations; thus, fluorescence measurements are always compared with a reference sample obtained by cleaving the biosensor using Tobacco Etch Virus (TEV) protease that produces a mixture of the two fluorescent proteins with an identical degree of maturation compared with the biosensor sample.

*Fluorescence spectra measurements*. Fluorescence emission spectra were recorded in a PTI (Photon Technology International) from 512 nm to 650 nm with excitation at 506 nm. in 50 mM phosphate buffer, 150 mM NaCl, 0.3 mM EDTA, 0.01% NaN_3_, pH 7.5 unless indicated otherwise.

*NMR experiments*. ^1^H-^15^N-HSQC NMR spectra were measured in a Bruker 600 Avance III instrument with a cryoprobe at 278 K and 298 K to observe the disordered regions and the SH3 domain, respectively. Assignments from the SNRE region in CLOBY and CLOBY-TEV were transferred directly from the previously assigned USH3 construct containing the SH4, unique and SH3 domains [22]. Chemical shift perturbations were monitored using the Farseer software [23].

*Dynamic Light Scattering*. Dynamic light scattering was measured at variable concentrations using a Malvern Zettasizer ZS90 in quartz cuvettes (Zen 2112). Before each measurement, the cuvette was washed with 5 mL of Hellmanex 5%, 5 mL of Acetic Acid 5%, 10 mL of water and then dried with 5 mL of 70% ethanol and compressed air.

Then, 70–80 µL of the sample were transferred to the cuvette. Samples were allowed to equilibrate at 25 °C for 120 s inside the instrument and the measurements were performed in 5 different runs, 10 replicates each. Temperature of the cuvette was always controlled by the instrument itself.

*Small-Angle X-ray Scattering*. SAXS data were measured in the SWING beamline of the Soleil synchrotron in a *Q* range from 0.04 to 5.6 nm^−1^ at 283 K at a wavelength of 1.0332 Å, with a sample to detector distance of 1.5 m. The coupled Size-Exclusion Chromatography SAXS (SEC-SAXS) mode was used for the measurement. Primary data reduction was carried out with Foxtrot [24], data processing was done with Chromixs [25], Guinier analysis was performed with Primus QT [26] and ensemble fitting was carried out with the ensemble optimization method [27].

*High-throughput fluorescence screening*. Ninety-six-well plates were measured with a Thermofluor FluoDiaT70 plate reader working at a constant temperature of 298 K. Excitation in the band 490–510 nm was achieved with a band-pass filter. Emission was measured at either 520–540 nm, including the emission maximum of mClover3 at 530 nm, or 580–600 that includes the emission maximum of mRuby3 at 590 nm. A total of 10% DMSO was present in ligand-containing and control samples. Possible response variations between wells were corrected by calibration with a solution of pure CLOBY. Matched plates containing CLOBY from the same preparation batch but treated with TEV protease (CLOBY-TEV) were used as a control to correct for possible differences in fluorophore maturation.

*Library preparation*. A customized commercial library of 1669 compounds was purchased from Selleckchem in 96-well plates containing 100 μL of 1 mM solution of each compound dissolved in DMSO. The entire library was aliquoted in plates retaining the original position of each compound using a Crystal Phoenix robot. One of the aliquots was diluted with DMSO to generate 100 μM stock solutions. For the initial screening, each well contained 100 μL of 1 μM CLOBY or CLOBY-TEV with or without 10 μM of ligand in 50 mM phosphate buffer, 150 mM NaCl, 0.3 mM EDTA, 0.01% NaN_3_, 10% DMSO, pH 7.5.

*Initial screening*. FRET results in increased emission of the acceptor (mRuby3) and decreased emission of the donor (mClover3). Thus, FRET changes can be detected at the two wavelengths. Assuming that most compound will not have an effect, possible hits were defined as those compounds causing an increase or a decrease in fluorescence intensity from the mean of the plate (88 compounds) that was larger than three standard deviations in the CLOBY-containing wells but was within three standard deviations from the mean in CLOBY-TEV containing wells. A second set of possible hits was selected using a weaker threshold of two standard deviations. Compounds causing very large changes in fluorescence were not considered in the calculation of the mean or the standard deviation and were individually studied using complete fluorescence spectra.

*Follow up screening*. Initial hits were analyzed in 96-well format using duplicated samples at three ligand concentrations (10 μM, 5 μM and 3 μM) and a CLOBY or CLOBY-TEV concentration 1 μM. Retained hits showed fluorescence intensity differences from the average control CLOBY larger than two standard deviations for at least two of the three concentrations while the fluorescence of the CLOBY-TEV in the presence of ligands remained within two standard deviations from the average fluorescence of ligand-free CLOBY-TEV at least at two concentrations.

## 3. Results

### 3.1. Biosensor Design

**Figure 1:**
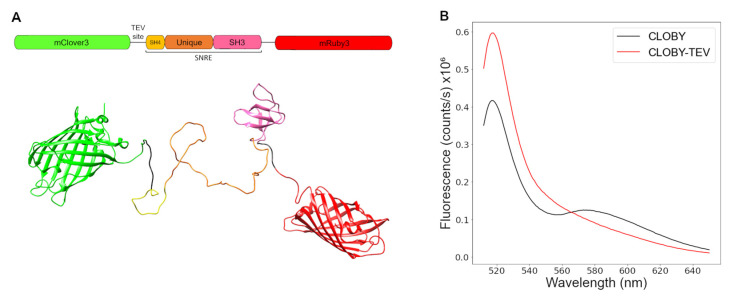
(**A**) Domain architecture and cartoon representation of CLOBY. (**B**) Fluorescence emission spectra of intact CLOBY (blue) and after treatment with TEV protease (red). Excitation wavelength was 506 nm and protein concentration was 1 µM.

Figure 1A shows a schematic representation of the CLOBY biosensor. The fluorescent proteins mClover3 and mRuby3 were chosen as they form an optimal FRET pair with a Förster radius of 6.5 nm, high photostability and brightness [19]. mClover 3 with an N-terminal histidine tag to facilitate protein purification and a spacer at its C-terminus was fused to the SH4 domain. mRuby3 was fused to the C-terminus of the SH3 domain through a flexible spacer. A TEV protease cleavage site was included in the spacer between mClover3 and the SH4 domain to provide a control with an identical mixture of the two disconnected fluorescent proteins for each preparation of the biosensor. This is important as a recurring problem when using fluorescent proteins is the slow maturation of the chromophore that starts after the protein is folded. Maturation of red fluorescent proteins can be especially slow introducing a possible reproducibility problem [28]. The TEV-treated control contains a control mixture of proteins with the same maturation level as the matching CLOBY sample.

### 3.2. TEV Treatment Confirms Intramolecular FRET at Low Concentrations

Fluorescence spectra were acquired at 1 μM concentration with excitation at 506 nm that corresponds to the absorbance maximum for mClover3. Two maxima were observed in the fluorescence emission spectra, corresponding to the emission of mClover3 at 518 nm and mRuby3 between 580 and 600 nm indicating FRET between the two fluorescent proteins fused at the ends of the SNRE (Figure 1B). The emission maximum of isolated mRuby3 is at 592 nm. Energy transfer from mClover3 (the donor) to mRuby3 (the acceptor) results in a decreased direct fluorescence of the donor. When FRET is prevented, the fluorescence of the donor increases, simultaneously with the decrease in the emission of the acceptor. A marked decrease in FRET is observed after treatment with TEV protease that separates the two fluorescent proteins, confirming its intramolecular origin.

### 3.3. Biosensor Characterization

#### 3.3.1. NMR Confirms That the SNRE Is Preserved in the CLOBY Biosensor

**Figure 2:**
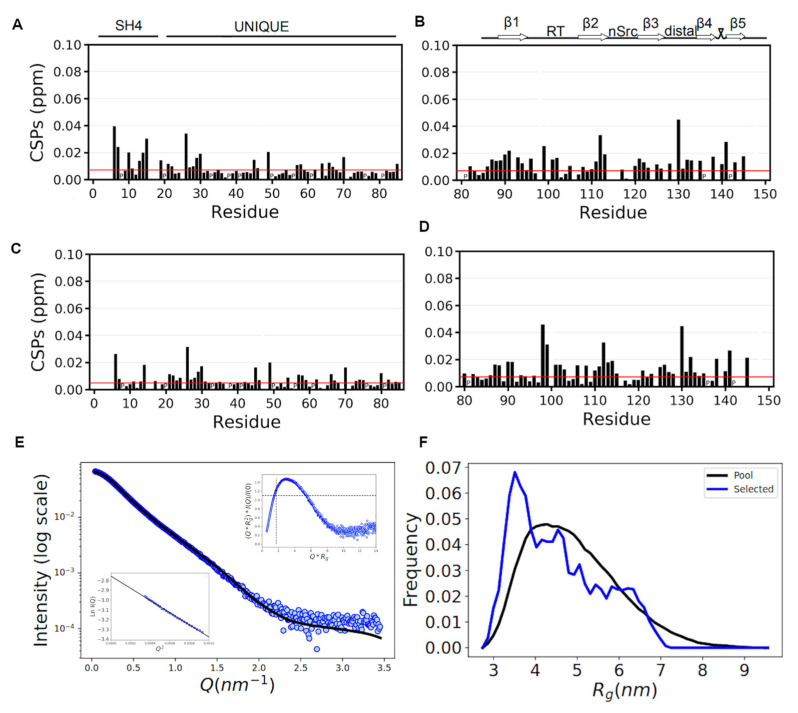
NMR chemical shift perturbation of CLOBY (**A**,**B**) and TEV-treated CLOBY (**C**,**D**) with respect to the isolated SNRE region of Src without the fluorescent proteins. Chemical shift differences were measured at 278 K for the disordered regions (**A**,**C**) and at 298 K for the SH3 domains (**B**,**D**). Red lines represent five standard deviations from the mean of the lowest 10% CSD. SAXS data of CLOBY are presented in panel (**E**). The main figure is the intensity profile, while the inserts represent a normalized Kratky plot (upper right) and a Guinier plot (lower left). Panel (**F**) presents the R_g_ distribution of the EOM-selected subensembles that best reproduce experimental SAXS data (blue) compared with the R_g_ distribution of the initial pool (in black).

We first addressed the question if the fluorescent proteins had a direct impact on the conformation of the disordered region of the SNRE. To answer this question, we used ^1^H-^15^N-Heteronuclear Single Quantum Correlation (HSQC) spectra to measure the chemical shifts of the NH nuclei of each of the residues and compare those of the SNRE part with a reference construct without the fused fluorescent proteins. Chemical shifts are very sensitive to even minor perturbations in the environment and interactions affecting individual residues. Combined chemical shift differences of the proton and nitrogen nuclei between CLOBY and the reference protein are presented in Figure 2. Residues in the disordered region show only minor perturbations confirming that the relevant interactions in the fuzzy complex are conserved. The largest perturbations occur near the point where the fluorescent protein is attached. A cluster of slightly perturbed residues is located between residues 21 and 30 with the largest perturbation affecting histidine 25 and glycine 26. Histidine 25 is extremely sensitive to experimental conditions, probably because its protonation state can be affected by the proximity of other charged residues in the fuzzy complex. Interestingly, this region has the same chemical shifts in CLOBY and the TEV-cleaved control, indicating that the observed perturbations are not due to the presence of mClover3.

The globular SH3 domain is more affected than the disordered regions by the presence of the fluorescent proteins, although most of the combined chemical shift perturbations are below 0.02 ppm. The largest perturbations correspond to the C-terminal region fused to mRuby3 although it also affects the N-terminus of the SH3 domain, which is structurally close. TEV-induced removal of mClover3 causes only minor changes in the SH3 domain, indicating that the observed perturbations are local. Therefore, we can conclude that the SNRE fuzzy complex is preserved in the CLOBY biosensor.

#### 3.3.2. SAXS Data Confirm That the Core of the Biosensor Remains Disordered

The small-angle X-ray scattering profile of CLOBY is consistent with that of a protein including globular as well as disordered domains [29] with a radius of gyration of 4.8 nm. The EOM [27] analysis suggests the protein is more compact than the reference random coil. A similar compaction was observed for a SNRE not fused to fluorescent proteins and was assigned to the formation of an intramolecular fuzzy complex [17].

#### 3.3.3. Transient Weak Self-Association Does Not Affect Intramolecular FRET

**Figure 3:**
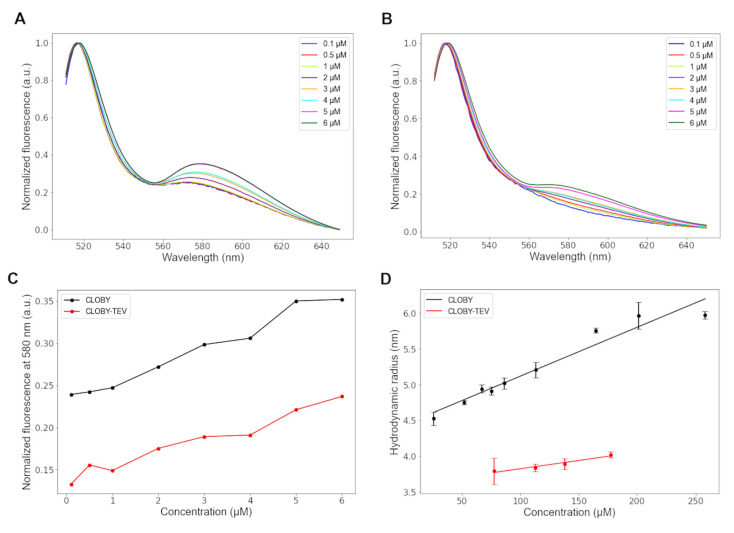
Concentration dependence of the normalized fluorescence of CLOBY (**A**) and TEV-treated CLOBY (**B**). The comparison of CLOBY and CLOBY-TEV presented in panel (**C**) highlights that the intermolecular FRET in the two samples is the same, and that intramolecular FRET dominates the fluorescence spectra at the 1 μM concentration used for screening experiments. The hydrodynamic radius measured by dynamic light scattering (**D**) at much higher concentrations (up to 275 μM for intact CLOBY) is also concentration dependent and provides an estimated dimer dissociation constant of 0.7 mM, confirming that self-association is negligeable at the working concentrations used in this study.

Apparent FRET changes may occur at high concentrations because a second acceptor molecule approaches the donor, or because two donor molecules approach each other, resulting in partial quenching of the direct fluorescence. This is indeed observed at high CLOBY concentrations (Figure 3). The hydrodynamic radius measured by dynamic light scattering shows a moderate linear increase with concentration indicating weak intermolecular interactions (Figure 3D). The hydrodynamic radius increases by around 33% at 275 μM, which corresponds to an apparent dimer dissociation constant of 0.7 mM.

A similar concentration dependent FRET is observed for intact CLOBY, where the two fluorescent proteins are part of the same molecule, and TEV-treated samples in which the concentration of the two fluorescent proteins is the same but they are in independent molecules. The fact that the concentration dependence of CLOBY and CLOBY-TEV is very similar suggests that the weak intermolecular interactions do not affect the average distance between mClover3 and mRuby3 in the same sensor that is sensitive to perturbations of the SNRE. However, to maximize intramolecular FRET, screening experiments were carried out at a constant biosensor concentration of 1 μM.

### 3.4. CLOBY Fluorescence Is Affected by Ligands Binding to the SNRE High-Throughput Screening of a Chemical Library Using the CLOBY Biosensor

**Figure 4:**
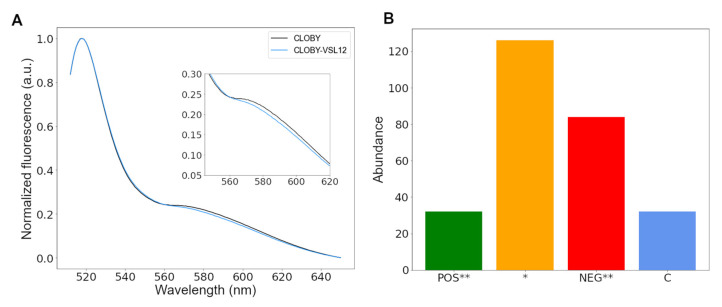
(**A**) CLOBY FRET is decreased in the presence of the VLS12 peptide, known to disturb the fuzzy complex of the SNRE. (**B**) Statistics of the first screening results based on fluorescence changes at 530 nm. Green and red bars represent the number of compounds increasing or decreasing the fluorescence by more than 3 standard deviations (indicated by **), respectively, only in intact CLOBY. The blue bar corresponds to colored compounds that could not be analyzed in the high-throughput assay. Orange bar corresponds to compounds with an effect (positive or negative) between one and two standard deviations (indicated by *).

The VSL12 peptide, with the sequence VSLARRPLPPLP, was originally discovered in a phage-display screen for sequences that bind to the c-Src SH3 domain [21]. Previous studies showed that the VSL12 peptide disturbs the fuzzy complex by competing with interactions of the unique domain with the RT-loop region of the SH3 domain [18]. Figure 4 shows the effect of adding an excess VSL12 on the fluorescence spectra of CLOBY. A small but clearly observable decrease in the emission at around 580 nm is observed. The effect of VSL12 on the fluorescence emission at 592 nm is larger than 6 times the standard deviation of a set of five measurement from independently prepared 1 μM CLOBY samples. Small effects are expected since the distance between the chromophores are affected only indirectly, through the perturbation of the conformational space of the intervening disordered region. The observed effect is consistent with the destabilization of the fuzzy complex increasing the distance between the reporter fluorescent protein.

**Figure 5:**
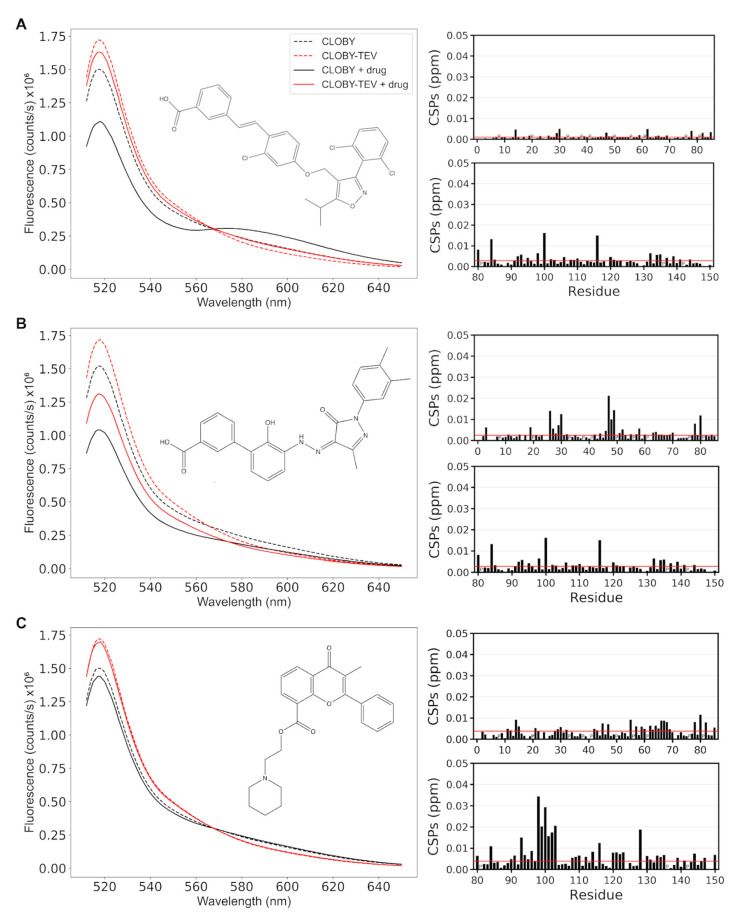
NMR validation of FRET results. Representative results of the three classes of hits defined by comparing fluorescence data and NMR perturbations. In each panel the fluorescence of CLOBY and CLOBY-TEV (at 1 μM concentration), in the presence or in the absence of excess drug (100 μM) are shown in the left. Chemical shift perturbations caused by the drug in the isolated SNRE, i.e., in the absence of the fluorescent proteins, are shown in the right. The effect on the disordered region (at 278 K) is shown on top and that that on the SH3 domain (at 298 K) are shown below. (**A**): GW4064; (**B**): Eltrombopag; (**C**): Flavoxate.

A custom designed library of 1669 compounds, including 1443 FDA-approved compounds and 226 anticancer compounds was used to test our biosensor. Although FRET changes can be detected either as a decrease in the intensity of the emission by the donor or an increase in the intensity by the acceptor (Figure 5A), the largest deviations were observed in the emission of mClover3. This is consistent with the largest spectral differences between CLOBY and CLOBY-TEV in the green region caused by FRET but may also reflect additional photophysical mechanisms. Although increases in the intensity around 520–540 nm most likely indicate a reduction in FRET, decreased emission of mClover3 may result either from an increased FRET or by partial quenching of the donor fluorescence. However, the fact that intensity changes are observed only in the intact biosensor but not in the TEV-cleaved control, allows the identification of drug-induced changes, although hits must be independently validated.

The distribution of hits is shown in Figure 4B. A total of 116 compounds caused changes larger than 3 standard deviations from the mean of each plate in CLOBY fluorescence but not in CLOBY-TEV (green and red bars, Figure 4B). Of these, 32 increased the emission around 530 nm, and 84 decreased it. A total of 126 compounds caused changes that were within one and three standard deviations from the mean (orange bar in Figure 4B) and 32 compounds caused large changes in the fluorescence spectra when added to CLOBY or CLOBY-TEV (blue bar, Figure 4B). Most of these compounds were intrinsically fluorescent and were analyzed individually.

The hits from the first screening were measured again at three concentrations (10 μM, 5 μM, and 3 μM) with two duplicates. Confirmed hits showed deviations in CLOBY fluorescence higher than two standard deviations in at least two of the concentrations, while at most showed deviations of this order in one of the concentrations in the CLOBY-TEV controls. Only 10 compounds were retained.

### 3.5. NMR Validation of Screening Hits

The 10 compounds from the second round of screening were validated by measuring the chemical shift perturbations induced in the NMR spectra of the isolated SNRE, i.e., the SH4–unique–SH3 domains of Src without the fluorescent proteins.

Three of the compounds that showed the largest changes in FRET showed no perturbation in the NMR spectra of the isolated SNRE. These compounds are Gossypol, a natural polyphenol isolated from cotton plant, 1,8-anthraquinone, and GW4064, a selective, non-steroidal farnesoid X receptor. Figure 5A shows the results from GW4064. The fluorescence spectra of CLOBY and CLOBY-TEV in the presence and in the absence of GW4064 cross in an isosbestic point, indicating that the compound is indeed causing a change between an open and closed state of the biosensor, but probably through a direct interaction with the fluorescent proteins. A possible explanation is that the drug can interact simultaneously with the two fluorescent proteins when they are part of the same fusion protein (in CLOBY) but not when they are in independent molecules (as in the CLOBY-TEV control).

The remaining 7 compounds showed perturbations in the chemical shift of residues in the unique domain. For six of them the most perturbed regions are in the highly flexible region between residues 26 and 30 and 46–49 (^26^GAGGG^30^ and ^46^GHRG^49^). The six compounds are: eltrombopag, used to treat thrombocytopenia; terbinadine, an antifungal drug; cornivaptan, an inhibitor of the vasopressin receptor; manidipine, an inhibitor of calcium channels; C-DIM12, a Nurr1 agonist stimulating apopotosis in bladder tumor and acting as neuroprotector in a Parkinson mouse model; and OSI-420, an inhibitor of the epithelial growth factor receptor. Data from eltrombopag are shown in Figure 5B. The fluorescence perturbation is weaker and no isosbestic point is observed, suggesting that the binding of the drug causes changes in the fuzzy complex that cannot be analyzed by a simple open–closed model. The fact that the chemical shift perturbations in this class of hits are always observed in the same region of the unique domain suggests that this is not the actual binding site but reflects the regions of the unique domain were the changes in the fuzzy complex cause the largest perturbation in the environment of the individual residues.

Finally, one of the compounds, flavoxate, another FDA-approved drug with anticholinergic effect, caused only a modest change in CLOBY fluorescence and minor changes in the NMR chemical shifts of the unique domain, but showed important perturbation in the SH3 domain, indicating a direct interaction with the RT loop, which is one of the main points of interaction of the disordered regions of Src with the SH3 domain. Interestingly, the 6 compounds that caused perturbations in the unique domain also caused minor perturbations in the RT loop, confirming that the compounds perturbed the fuzzy complex.

## 4. Discussion

Drug screening for intrinsically disordered targets is still a challenging task. The fuzzy complex present in the N-terminal region of c-Src is a regulatory region validated by the strong decrease in cancer invasion and proliferation caused by mutations in the intrinsically disordered unique domain. To find drugs modulating the Src fuzzy complex, we have designed a biosensor displaying FRET between fluorescent proteins and a screening protocol to check for drug-induced changes in FRET intensity that could be diagnostic of perturbations in the fuzzy complex.

Unimolecular FRET-based biosensors have been extensively used to monitor phosphorylation [30,31,32]. These sensors are based on a well-defined interaction between a specific phosphorylatable site and a phosphorylation recognition domain that causes a marked change in the distance between two fluorescent proteins forming a Förster pair. Specifically, Src biosensors have been developed to measure Src tyrosine kinase activity through the interaction of a Src substrate sequence and a SH2 domain increasing the distance between the fluorescent proteins [33]. Another Src FRET biosensor was designed to measure Src activation based on the proximity between the SH2 domain and the C-terminus [34]. A recently described Src biosensor is based on intermolecular FRET using a 7-mer peptide tag that is only exposed when Src is activated. When exposed, it can bind a reporter protein bringing a donor–acceptor fluorescent pair to a short distance [35]. However, these biosensors monitor the formation of a well-defined complex around the globular domains. CLOBY differs from these biosensors by focusing on the fuzzy complex of Src between the folded SH3 domain and the entire N-terminal disordered region.

Control experiments using NMR and SAXS confirm that CLOBY retains the fuzzy complex, as seen by the invariant NMR chemical shifts of residues that are diagnostic of the formation of the fuzzy complex in the presence and in the absence of the fused fluorescent proteins. Interestingly, SAXS results show a compaction of CLOBY with respect to the reference random coil ensemble, similar to what had been observed in Src [17].

The fusion protein approach employed here generates molecules with a stoichiometric two-color labeling. Alternative approaches for two-color labeling of the same molecule using organic dyes incorporated by reaction with cysteines or unnatural amino acids [36] require the use of a mixture of dyes, which generates a statistical mixture of proteins in which only 50% of the molecules contain different dyes and could give FRET, but even these FRET-active molecules are formed by a 50% mixture of positional isomers. Given the weak FRET responses observed, fusion proteins are probably the best approach.

The FRET responses caused by a known peptide ligand of the fuzzy complex, and by non-peptidic molecules identified by high-throughput screening, are much weaker than that of biosensors directed to the globular domain. This is not unexpected considering the fuzzy nature of the region being tested. The observed FRET changes depend on variations on the average end-to-end distance, and therefore are scaled down as compared with changes expected for a transition involving the release of a fixed closed conformation. For the moment, sensitivity is not enough for in vivo studies. However, our in vitro screening of a library of FDA-approved and anticancer compounds has identified promising hits.

Although the fluorescence screening provides the required high throughput, it does not identify the interaction site. Chemical shift NMR perturbation caused by the fluorescence-detected hits have confirmed that in seven out of the ten retained hits, these compounds perturb the unique or the SH3 domain in the absence of the fluorescent proteins, thus validating CLOBY as a biosensor for the identification of drugs interacting with the fuzzy complex in the Src N-terminal regulatory element.

## Data Availability

The data presented in this study are available on request from the corresponding author.
